# Metabolic Interactions of Purine Derivatives with Human ABC Transporter ABCG2: Genetic Testing to Assess Gout Risk

**DOI:** 10.3390/ph6111347

**Published:** 2013-11-04

**Authors:** Toshihisa Ishikawa, Wanping Aw, Kiyoko Kaneko

**Affiliations:** 1 RIKEN Center for Life Science Technologies, 1-7-22 Suehiro-cho, Tsurumi-ku, Yokohama 230-1145, Japan; 2 Graduate School of Biomedical Science, Tokyo Medical Dental University, 1-5-45 Yushima, Bunkyo-ku, Tokyo 113-8510, Japan; 3 Laboratory of Biomedical and Analytical Sciences, Faculty of Pharma Sciences, Teikyo University, 2-11-1 Kaga, Itabashi-ku, Tokyo 173-8605, Japan

**Keywords:** ABC transporter, ABCG2, gout, hyperuricemia, kidney, SNP, uric acid

## Abstract

In mammals, excess purine nucleosides are removed from the body by breakdown in the liver and excretion from the kidneys. Uric acid is the end product of purine metabolism in humans. Two-thirds of uric acid in the human body is normally excreted through the kidney, whereas one-third undergoes uricolysis (decomposition of uric acid) in the gut. Elevated serum uric acid levels result in gout and could be a risk factor for cardiovascular disease and diabetes. Recent studies have shown that human ATP-binding cassette transporter ABCG2 plays a role of renal excretion of uric acid. Two non-synonymous single nucleotide polymorphisms (SNPs), *i.e.*, 421C>A (major) and 376C>T (minor), in the *ABCG2* gene result in impaired transport activity, owing to ubiquitination-mediated proteosomal degradation and truncation of ABCG2, respectively. These genetic polymorphisms are associated with hyperuricemia and gout. Allele frequencies of those SNPs are significantly higher in Asian populations than they are in African and Caucasian populations. A rapid and isothermal genotyping method has been developed to detect the SNP 421C>A, where one drop of peripheral blood is sufficient for the detection. Development of simple genotyping methods would serve to improve prevention and early therapeutic intervention for high-risk individuals in personalized healthcare.

## 1. Introduction

In our body, nutrients and xenobiotics are not only enzymatically metabolized, but also transported by various transporters including members of the solute carrier (SLC) superfamily and some ATP-dependent efflux pumps of the ATP-binding cassette (ABC) superfamily [1]. Transporter proteins mediating the uptake and efflux of metabolites into and out of cells are key determinants of the *in vivo* distribution and elimination of a variety of endogenous substances and xenobiotics. The human ATP-binding cassette (ABC) protein family involves a total 48 different genes, and they are classified into seven sub-families (from A to G) [2]. While ABCG2 was originally identified as a multi-drug efflux pump in drug-resistant breast cancer MCF-7/AdrVp cells [3], recent studies have shown that ABCG2 is an important human uric acid transporter in the kidney [4]. Genetic polymorphisms (421C>A and 376C>T) in the *ABCG2* gene have been identified to cause hyperuricemia and gout [4,5]. In this review article, we provide an overview on the physiological role of human ABC transporter ABCG2 in purine metabolism and propose genotyping-based “personalized healthcare” to assess gout risk.

## 2. Purine Metabolism

**Figure 1:**
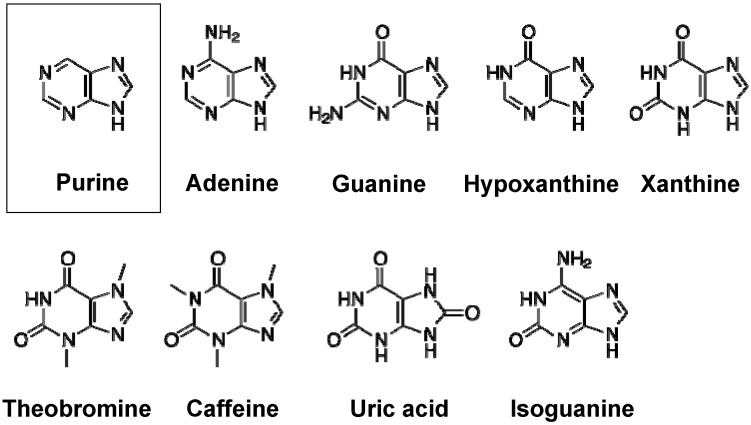
Chemical structures of purine and its derivatives, *i.e.*, adenine, guanine, hypoxanthine, xantine, theobromine, caffeine, uric acid, and isoguanine.

Purines are components of nucleosides, the building blocks of DNA and RNA. Purine nucleosides, *i.e.*, adenosine and guanine, are used in the creation of other metabolically important factors as well, such as ATP, GTP, cyclic AMP, S-adenosylmethionine, nicotinamide adenine dinucleotide (NADH), and nicotinamide adenine dinucleotide phosphate (NADPH). The family of purine and its derivatives includes adenine, guanine, isoguanine, hypoxanthine, xanthine, theobromine, caffeine, and uric acid (Figure 1).

Given the importance of purine-containing molecules for survival, vertebrates, including humans, have developed robust systems for synthesizing sufficient purine nucleosides for their metabolism using readily available materials (such as glucose, glycine, and glutamine), as well as recycling purine nucleosides from throughout the body or from the diet.

**Figure 2:**
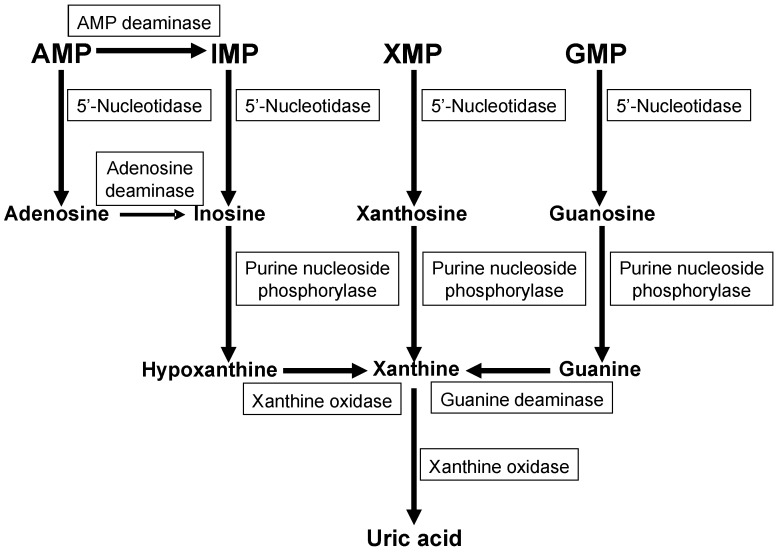
Metabolic pathways of uric acid formation from nucleotide monophosphates. AMP, adenosine monophosphate; IMP, inosine monophosphate; XMP, xanthine monophosphate, GMP, guanine monophosphate.

In mammals, excess purine nucleosides are removed from the body by breakdown in the liver and excretion from the kidneys. For most mammals, the purines are first converted into the intermediate uric acid, which is then metabolized by the enzyme uricase into the compound allantoin. Allantoin is a very soluble compound that can easily travel through the bloodstream, become filtered by the kidneys, and be excreted from the body. In contrast to other mammals, humans and primates lack a functional uricase enzyme, and can only break purines down into uric acid. Figure 2 depicts the metabolic pathways of purine catabolism and uric acid formation. The metabolic pathways consist of multiple steps of reactions catalyzed by various enzymes, such as AMP deaminase, adenosine deaminase, 5′-nucleotidase, purine nucleoside phosphorylase, guanine deaminase, and xanthine oxidase. In humans, uric acid is the final product of purine metabolism.

## 3. Renal Excretion of Uric Acid

The levels of uric acid in the blood depend on two factors. The first is the rate of uric acid synthesis in the liver. Since uric acid results from purine degradation, its levels are influenced by both the amount of purines synthesized in the body, as well as the amounts of purines absorbed from the diet [6]. The second determinant of blood uric acid levels is the rate of uric acid excretion from the kidneys. Excretion has the greatest effect on blood uric acid levels, with about 90% of hyperuricemia cases attributed to impaired renal excretion [7].

It is suggested that hyperuricemia increases the risk of not only gout, but also other diseases as well, including hypertension, kidney disease, and metabolic syndrome [8]. Even during the asymptomatic periods between gout attacks, the body is exposed to periods of low-grade, chronic inflammation. The propensity for excessive blood uric acid and gout can also be increased by other disease states [9]. It is important, therefore, to prevent the onset of gout. Early therapeutic intervention is needed for high-risk individuals.

**Figure 3:**
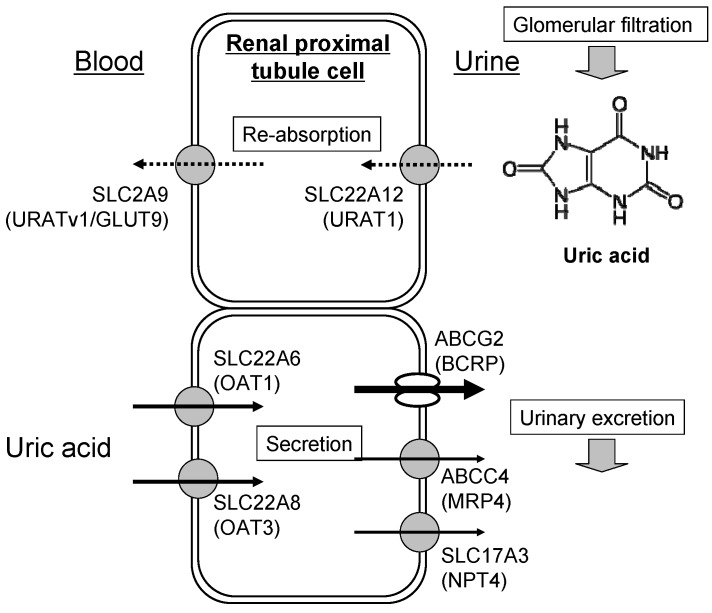
Schematic illustration of renal re-absorption and excretion of uric acid. Uric acid filtrated from the renal glomerular body is first re-absorbed by human renal proximal tubule cells. Thereafter, uric acid is eliminated, in part, from the blood circulation to urine by renal transporters. Arrows in the figure indicate the direction of transport of uric acid. Dotted and solid lines indicate re-absorption and secretion, respectively.

Impaired excretion is most often due to abnormalities in uric acid transporters expressed in the kidney [4,10,11]. Uric acid transporters control the movement of uric acid through renal proximal tubules, as shown in Figure 3. The physiological relevance of uric acid transporters in humans is established by genetic variation causing hyper-/hypo-uricemia and gout. SLC22A12 (URAT1) and SLC2A9 (URATv1/GLUT9) are major players in the re-absorption of uric acid into blood [12,13,14,15,16,17], whereas SLC22A6 (OAT1), SLC22A8 (OAT3), SLC17A3 (NPT4), ABCC4 (MRP4), and ABCG2 (BCRP) are involved in the renal secretion of uric acid into urine [4,18,19,20,21].

## 4. Cause of Gout and Therapeutics

Gout is one of the oldest known and most common forms of arthritis; it is a crystal deposition disease in which crystals of monosodium urate form in joints and other tissues [22,23]. Gout usually involves sudden attacks of severe pain, often in the joint at the base of the big toe and frequently in the wee hours of the morning, when body temperature is lowest. Gout attacks cause a characteristic painful inflammation of one or more joints of the extremities, or nodules in soft tissues called tophi. An acute attack of gout, although brief and usually subsiding spontaneously, can be temporarily debilitating, and predisposes an individual to subsequent attacks.

For therapeutic intervention of gout, several drugs were developed and are widely used. Xanthine oxidase inhibitors, for example, reduce the activity of xanthine oxidase, the final step in uric acid synthesis. This has the effect of lowering uric acid production. Allopurinol (Zyloprim) has a long history of usage as a xanthine oxidase inhibitor; recently febuxostat (Uloric) has been approved for treatment of hyperuricemia in the US. Febuxostat exhibits greater uric acid-lowering effects than allopurinol, although the incidence of gout flares is similar between the two drugs [24]. Uricosuric drugs are also often used to reduce uric acid level in the systemic blood circulation, primarily by reducing the re-absorption of uric acid from the kidneys back into the blood. Probenecid (Benemid) and sulfinpyrazone (Anturane) are two examples. These drugs, however, tend to increase urinary uric acid levels, which can cause kidney stones. Recently, the outside and inside of the kidney stones were measured non-destructively with a micro area X-ray diffractometer [25]. Proteins, such as uromodulin and albumin, are often detected in stones regardless of crystal components, where uromodulin is the most abundant urinary glycoprotein. Furthermore, immunoglobin G fragments were also detected in uric acid stones [25]. In some persons with loss-of-function mutations of URAT1, uricosurics such as benzbromarone and losartan had no effect, suggesting these drugs act on URAT1 *in vivo* [26].

## 5. Genetic Analysis of Gout Risk

**Figure 4:**
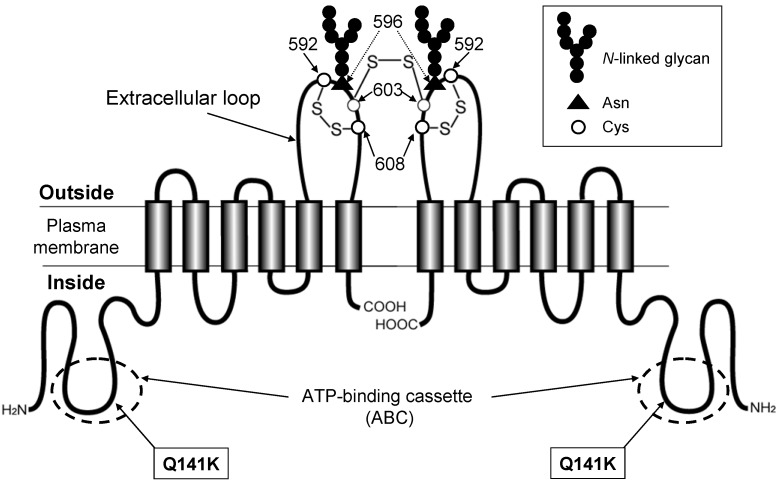
Schematic illustration of human ABCG2 and its non-synonymous polymorphisms. The ABCG2 protein expressed in the plasma membrane is a homodimer linked via a cysteinyl disulfide bond. The cysteine residue corresponding to Cys603 of human ABCG2 is involved in the homodimer formation, whereas Cys592 and Cys608 form an intra-molecular disulfide bond that is important for *N*-linked glycan formation at Asn596. The SNP 421C>A is a non-synonymous polymorphism that leads to amino acid substitution; Gln to Lys (Q141K) in the intracellular loop containing an ATP-binding cassette (ABC).

Recently, large meta-analyses of GWAS have revealed that SNPs in the *SLC2A9* (GLUT9) and *ABCG2* genes are strongly associated with the phenotype of gout [27,28,29]. Since serum levels of uric acid are highly heritable, the involvement of genetic factors in gout was previously speculated. Several laboratories have independently found that the SNP 421C>A in the *ABCG2* gene (Figure 4) is one of the major genetic factors for elevated serum uric acid levels and the increased risk of gout [4,5,30].

**Figure 5:**
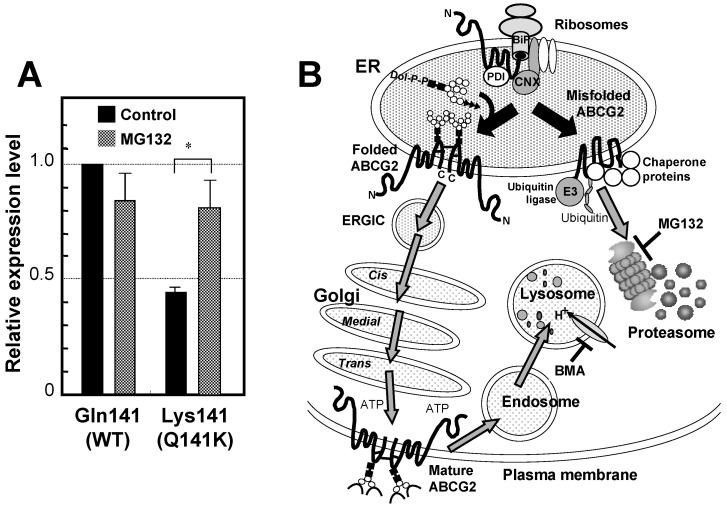
Effect of the SNP variant (Q141K) on the protein expression level and degradation of ABCG2. (**A**) The ABCG2 wild type (WT) protein has glutamine residue at amino acid position 141. To assess the effect of Q141K variant on the protein expression level, Flp-In-293 cells expressing WT and the Q141K variant were incubated in the absence or presence of MG132 (2 μM) for 24 h. ABCG2 WT and Q141 variant proteins were analyzed by immunoblotting with the ABCG2-specific monoclonal antibody (BXP-21) after PNGase F treatment. The glyceraldehyde-3-phosphate dehydrogenase (GAPDH) protein level was analyzed by GAPDH-specific antibody. The signal intensity ratio (ABCG2/GAPDH) was normalized to the control level. Data are expressed as means ± S.D. in triplicate experiments [33]. (**B**) Correctly processed ABCG2 WT protein is destined to reach the plasma membrane and is then degraded by the endosome-lysosome pathway after remaining in the plasma membrane domain for a certain period. In contrast, the ABCG2 Q141K variant protein is recognized as a misfolded form and then undergoes ubiquitination-mediated proteasomal degradation. Bafilomycin A_1_ (BMA) and MG132 inhibit lysosomal and proteasomal degradation, respectively.

ABCG2 expressed on the apical side of the proximal tubular cells in human kidney plays a pivotal role in renal excretion of serum uric acid. Introduction of the mutation Q141K encoded by the common SNP (rs2231142) by site-directed mutagenesis resulted in 53% reduced urate transport rates compared to wild-type ABCG2 (*p* < 0.001). The expression levels of the Q141K variant are reduced by ubiquitin-mediated proteasomal degradation [31,32,33,34] (Figure 5). Thus, renal excretion of serum uric acid via ABCG2 is impaired in persons who are carrying the 421A allele (Q141K variant). As a consequence, serum uric acid levels are elevated, which enhances the risk of gout.

## 6. Genetic Polymorphisms in *ABCG2* Gene

The *de novo* synthesized ABCG2 protein undergoes the formation of inter- and intramolecular disulfide bonds in the lumen of endoplasmic reticulum (ER) by the action of protein disulfide isomerase and FAD-bound ER oxidoreductin 1 [34,35] (Figure 5B). The ABCG2 protein is then *N*-glycosylated at Asn596. *N*-linked glycans are added *en block* to proteins as “core oligosaccharides” (Glc_3_Man_9_GlcNAc_2_). Calnexin (CNX) is located near the translocon and can interact with nascent peptide chains of *N*-linked glycosylated proteins. After *N*-glycosylation, ABCG2 protein is transferred to the *Golgi* for further processing. The correctly processed ABCG2 protein is finally destined to the plasma membrane and then degraded by the endosome-lysosome pathway after remaining in the plasma membrane domain for a certain period. In contrast, the misfolded ABCG2 protein undergoes ubiquitination-mediated proteasomal degradation. Bafilomycin A_1_ (BMA) and MG132 inhibit lysosomal and proteasomal degradation, respectively.

**Figure 6:**
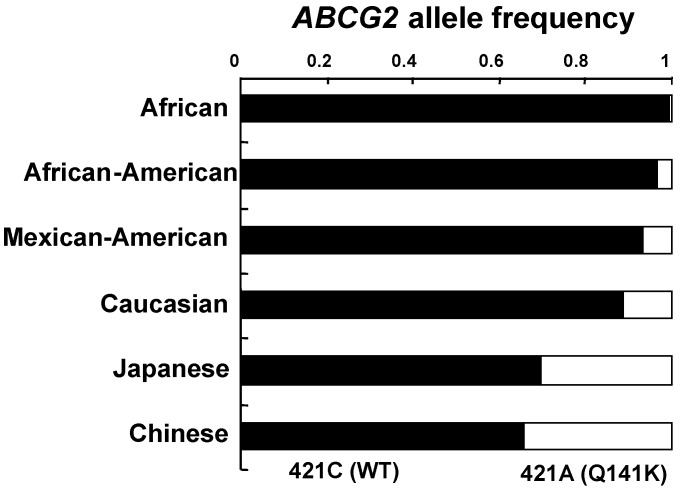
The allele frequencies of 421C (WT) and 421A (Q141K) among different ethnic populations. Data are calculated from Ishikawa *et al*. [36].

Sequencing of the *ABCG2* gene from human samples has revealed over 80 different, naturally occurring sequence variations. [36,37,38,39,40,41,42,43,44,45,46]. Amongst them, SNP 421C>A polymorphism located in exon 5 leads to the replacement of the glutamine residue with a positively charged lysine residue. This polymorphism affects the ATP-binding domain, between the Walker A motif (amino acid residues 83–89) and the signature region (amino acid residues 186–189). This SNP variant has also been detected in all ethnic groups tested: the allele frequency ranged between 0% and 35%, (North-Saharan Africans, sub-Saharan Africans, and African-American subjects with low; and Japanese and Chinese populations with high allele frequencies) [36,39,40,43,44] (Figure 6).

An investigation of the expression level of ABCG2 in 99 Japanese placenta samples revealed that individuals homozygous for the Q141K variant showed significantly lower expression levels of this transporter protein, while the heterozygous samples displayed intermediate expression levels [44]. Based on those observations, it is assumed that the protein stability of the Q141K variant is significantly reduced without showing significant changes in its mRNA levels. Evidence has been provided to show that the Q141K variant of ABCG2 is degraded via ubiquitin-mediated proteasomal degradation [31,32,33,34,35]. The level of the Q141K variant protein could be recovered when proteosomal degradation was inhibited by MG132 *in vitro* (Figure 5A). The Q141K mutation does not interfere with the nucleotide-binding domain/intracellular loop interactions but greatly affect the protein-protein interactions necessary for the dimerization of ABCG2. Two amino acid residues, *i.e.*, Lys-473 (K473) and Phe-142 (F142), were found to play a pivotal role in dimerization of ABCG2 protein [47].

In addition to the Q141K variant, a minor SNP in exon 4, 376C>T, substituting a stop codon for Gln126, was found in the Japanese population with an allele frequency of 2.4% [37]. It has been postulated that the 376C>T SNP may have a high impact, since active ABCG2 protein will not be synthesized from the variant allele. The variant Q126stop (376C>T) was consistently observed in certain Japanese cohorts; however, it was absent in Caucasian and African-American groups [39,41,44]. Matsuo *et al*. [4] reported that the genotype combinations of Q126stop and Q141K are clinically important biomarkers to predict the possible risk of gout in the Japanese population.

In the US, approximately three million individuals are suffering from often insufficiently treated gout. Woodward *et al*. [30] performed a population-based study with 14,783 individuals to examine SNP 421C>A (rs2231142) as the causal variant. Highly significant associations were found between uric acid levels in blood and the incidence of gout (adjusted odds ratio 1.68). Their data suggest that at least 10% of all gout cases in Caucasians are attributable to SNP 421C>A (Q141K).

## 7. Rapid Genotyping to Assess Gout Risk

**Figure 7:**
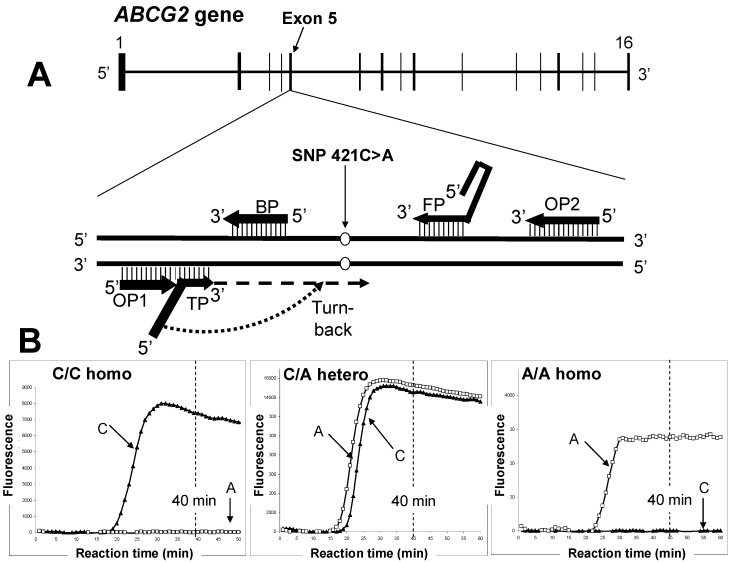
Rapid genetic testing to assess gout risk by the SmartAmp method. (**A**) Schematic illustration of the human *ABCG2* gene located on chromosome 4q22. The SNP 421C>A resides in exon 5. (**B**) Detection of SNP 421C>A in the human *ABCG2* gene by the SmartAmp method. Three panels depict the time-courses of the SmartAmp assay reactions with *ABCG2* allele–specific primers carrying WT (421C) or SNP (421A) alleles; namely, C/C homozygote, C/A heterozygote, and A/A homozygote. After genomic DNA in peripheral blood samples (5 μL) was denatured at 98 °C for 3 min, the genotyping reactions were allowed to proceed isothermally at 60 °C for 60 min in a Mx3000P PCR system (Agilent Technologies, Santa Clara, CA, USA) [48]. DNP amplification was continuously monitored by detecting the fluorescence of DNA-intercalated SYBR Green I dye in the reaction mixture.

Genetic diagnostics is a growing field that is gradually becoming more user-friendly with the introduction of portable devices and faster nucleic acid detection. Figure 7 shows the detection of SNP 421C>A in the *ABCG2* gene by the SmartAmp method. This method was developed based on the principal concept that DNA amplification itself is the signal for detection of a genetic mutation or SNP. Differing from the widely-used PCR, the SmartAmp method consists of both isothermal DNA amplification and SNP-discriminating reactions [48].

In the SmartAmp method, the entire DNA amplification process requires five primers: turnback primer (TP), boost primer (BP), forward primer (FP), and two outer primers (OP1 and OP2) (Table 1). Primers are selected based on the SmartAmp primer algorithm by using the optimal melting temperature and product size range. The genomic sequence between the annealing sites of the TP and FP primers is the target region that will be amplified by the reaction (Figure 7).

**Table 1:** Primer sets to detect WT and SNP alleles in the human *ABCG2* gene.

WT (421C)-detection Primer Set	
**Primer**	**Sequence (5'→3')**
TP	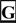 TAAGTTTTCCTTAAGGATGATGTTGTG
FP	ACCTTCTGTACCCTCAGAAGGTGCCGAAGAGCTGCTGAGAAC
BP	ACCGTCAGAGTGCCCAT
OP1	TTATCATTATGTCTCATT
OP2	ATGATTCGTCATAGTTGT
**SNP (421A)-detecting Primer Set**	
**Primer**	**Sequence (5'→3')**
TP	T 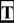 TAAGTTTTCTCTTAAGGATGATGTTGTG
FP	ACCTTCTGTACCCTCAGAAGGTGCCGAAGAGCTGCTGAGAAC
BP	ACCGTCAGAGTGCCCAT
OP1	TTATCATTATGTCTCATT
OP2	ATGATTCGTCATAGTTGT

DNA polymerase reaction proceeds from the 3′-end of TP primer (see Figure 7), and the turn-back region (underline) of TP primer recognizes the nucleotide (C or A) corresponding to the position 421 in the newly synthesized DNA strand. The C-or-A recognizing nucleotide (G or T) is marked by a square in the sequence of each TP primer. The molar ratio of primers in the genotyping reaction mixture was TP:FP:BP:OP1:OP2 = 8:8:4:1:1.

In the SmartAmp method, clinical samples are processed by using the enzyme *Aac* polymerase. This enzyme is highly resistant to cellular contaminants and hence works directly on blood samples, following a simple heat treatment (98 °C, 3 min) to degrade RNA and denature proteins [48]. This is a great advantage of the SmartAmp method over the commonly used PCR-based techniques with *Taq* DNA polymerase, which is easily inhibited by impurities. The application of SmartAmp for practical diagnostics should be evaluated according to the principle of amplification *versus* non-amplification with respect to threshold values. The amount of DNA-intercalating SYBR Green I dye accumulated during the reaction is monitored, and thereby SNP typing can be determined by referring to the intensity of the non-amplification fluorescence.

Recently Matsuo *et al*. examined their hypothesis of whether common dysfunction of ABCG2 causes early-onset gout [49]. They genotyped a total of 705 Japanese male gout patients with onset age data as well as 1,887 male controls. ABCG2 functions were estimated based on genotype combinations (421C>A and 376C>T). They found that the onset age was 6.5 years earlier with severe ABCG2 dysfunction than with normal ABCG2 function (*p* = 6.14 × 10^−3^). Patients with mild to severe ABCG2 dysfunction accounted for 88.2% of early-onset cases (twenties or younger). Severe ABCG2 dysfunction particularly increased the risk of early-onset gout (odds ratio 22.2, *p* = 4.66 × 10^−6^). Based on those findings, Matsuo *et al*. conclude that common dysfunction of ABCG2 is a major cause of early-onset gout [49]. Thus, genotyping of the *ABCG2* gene would serve to improve prevention and early therapeutic intervention for high-risk individuals.

## 8. Conclusions

Nowadays, pharmacogenomics is widely used in predicting drug efficacy and drug-induced adverse reactions [50]. This knowledge on the effects of genetic polymorphisms is applicable in drug discovery and development as well as in the clinical use of drugs. Increases in efficacy and safety by the individualization of medical treatment may have benefits in financial terms, if information is presented to show that personalized medicine will be cost-effective in healthcare systems. In fact, several genetic polymorphisms in drug metabolizing enzyme genes (e.g., *CYP2C9*, *CYP2C19*, *CYP2D6*, *TPMT*, *VKORC1*) and transporter genes (e.g., *SLCO1B1* and *ABCG2*) are on the list of genetic testing in personalized medicine. As described in this article, the SNPs of 421C>A and 376C>T in the human *ABCG2* gene are recognized as clinical biomarkers to assess hyperuricemia and gout.

The rapid growth of personalized medicine is being supported by emerging new technologies together with accumulating knowledge of pharmacogenomics. Basic technologies of molecular diagnostics play a role in expanding pharmacogenomic information, particularly with respect to SNP genotyping. Diagnosis is thus integrated with therapy for selecting treatments as well for monitoring results. Cost-effective methods should be developed for genotyping, however, and it would be desirable to include this information in the patient’s record as guidance for physicians to individualize the treatment. The accurate measurement of allele frequency variations among population groups with different sensitivities to diseases and/or different responses to drugs is fundamental to genetic epidemiology. Development of personalized medicine, including point-of-care technology, requires the integration of various segments of biotechnology, clinical medicine, and pharmacology. Multiple players could promise the realization of personalized medicine and personalized healthcare.
